# Automatic Left Ventricle Segmentation from Short-Axis Cardiac MRI Images Based on Fully Convolutional Neural Network

**DOI:** 10.3390/diagnostics12020414

**Published:** 2022-02-05

**Authors:** Zakarya Farea Shaaf, Muhammad Mahadi Abdul Jamil, Radzi Ambar, Ahmed Abdu Alattab, Anwar Ali Yahya, Yousef Asiri

**Affiliations:** 1 Faculty of Electrical and Electronic Engineering, Universiti Tun Hussein Onn Malaysia, Parit Raja, Batu Pahat 86400, Johor, Malaysia; aradzi@uthm.edu.my; 2 Department of Computer Science, College of Science and Arts, Sharurah, Najran University, Najran 61441, Saudi Arabia; aaalattab@nu.edu.sa; 3 Department of Computer Science, Faculty of Computer Science and Information Systems, Thamar University, Dhamar 87246, Yemen; aaesmail@nu.edu.sa; 4 Department of Computer Science, College of Computer Science and Information Systems, Najran University, Najran 61441, Saudi Arabia; yasiri@nu.edu.sa

**Keywords:** left ventricle segmentation, cardiac short-axis MRI, fully convolutional network, pixel weights balancing, medical image processing

## Abstract

Background: Left ventricle (LV) segmentation using a cardiac magnetic resonance imaging (MRI) dataset is critical for evaluating global and regional cardiac functions and diagnosing cardiovascular diseases. LV clinical metrics such as LV volume, LV mass and ejection fraction (EF) are frequently extracted based on the LV segmentation from short-axis MRI images. Manual segmentation to assess such functions is tedious and time-consuming for medical experts to diagnose cardiac pathologies. Therefore, a fully automated LV segmentation technique is required to assist medical experts in working more efficiently. Method: This paper proposes a fully convolutional network (FCN) architecture for automatic LV segmentation from short-axis MRI images. Several experiments were conducted in the training phase to compare the performance of the network and the U-Net model with various hyper-parameters, including optimization algorithms, epochs, learning rate, and mini-batch size. In addition, a class weighting method was introduced to avoid having a high imbalance of pixels in the classes of image’s labels since the number of background pixels was significantly higher than the number of LV and myocardium pixels. Furthermore, effective image conversion with pixel normalization was applied to obtain exact features representing target organs (LV and myocardium). The segmentation models were trained and tested on a public dataset, namely the evaluation of myocardial infarction from the delayed-enhancement cardiac MRI (EMIDEC) dataset. Results: The dice metric, Jaccard index, sensitivity, and specificity were used to evaluate the network’s performance, with values of 0.93, 0.87, 0.98, and 0.94, respectively. Based on the experimental results, the proposed network outperforms the standard U-Net model and is an advanced fully automated method in terms of segmentation performance. Conclusion: This proposed method is applicable in clinical practice for doctors to diagnose cardiac diseases from short-axis MRI images.

## 1. Introduction 

Cardiovascular disease is regarded as one of the most severe threats to human health, and it has contributed to an increase in the global mortality rate. According to the World Health Organization, 17.9 million people died from cardiovascular disease in 2016, accounting for 31% of worldwide deaths [1]. As a result, there is a growing emphasis on research and technologies that can effectively improve the diagnosis of cardiovascular diseases while also lowering the mortality rate caused by those diseases. In recent years, the diagnosis of cardiovascular diseases has become more accessible thanks to advancements in medical imaging techniques such as computed tomography (CT) and cardiac magnetic resonance imaging (CMRI).

**Figure 1:**
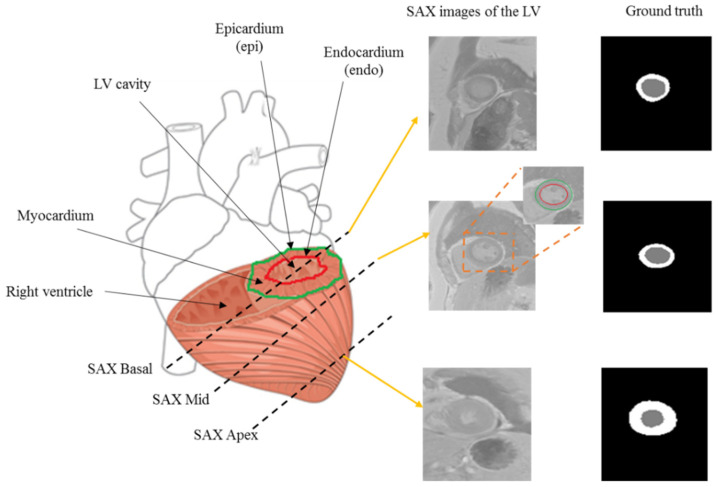
LV short-axis MRI and corresponding ground truth (labels).

MRI is one of the most regularly utilized medical imaging modalities for diagnosing cardiovascular disease because it is non-invasive and produces high-resolution images. Segmentation of cardiac short-axis MRI is critical for quantifying cardiac function by analyzing clinical metrics such as ventricular volumes, stroke volumes, and myocardium in the early detection of cardiovascular diseases. Segmentation of the LV is vital for accurate assessments of cardiac function indicators such as ejection fraction, LV volume, and LV mass, all of which are important in diagnosing cardiovascular diseases [2,3,4]. To better understand the LV segmentation task, Figure 1 shows short-axis (SAX) images of the LV at the basal, middle, and apical slices along with their corresponding ground truth (labels). The primary goal of LV segmentation is to delineate the LV’s contours (epicardium and endocardium). On the other hand, manual LV segmentation is a time-consuming and error-prone task for medical experts. Therefore, a fully automated LV segmentation method from the short axis is urgently needed.

For LV segmentation, a variety of techniques have been proposed. Active contour, level set, and graph cut are examples of model-based approaches. These models, on the other hand, are semi-automatic and rely heavily on a successful initialization step. Deep learning-based algorithms have become frequently used in medical image segmentation due to rapid advancements in computer hardware and the availability of massive training data. Convolutional neural networks (CNNs), a standard deep learning-based method, have recently achieved excellent results in various computer vision fields, including object detection [5], image classification [6], and image segmentation [7]. Following this trend, several CNN-based techniques for LV segmentation have been proposed [8,9,10,11,12,13,14,15] and have shown promising results in clinical practice. However, accurate segmentation of the LV and myocardium from cardiac MRI remains a challenge in clinical practice for several reasons, including changes in the LV morphology across slices, an imbalance in pixels between the LV area and the background, and incorrect pixel representation for the target area. Furthermore, Xiong et al. [16] complained that deep learning methods are data-driven and need a massive amount of data for training, and the available labeled dataset of the LV is small. Thus, a small labeled data results in poorer performance when utilizing deep learning approaches. 

The ability to develop and compare the performances of FCN models is based on several conditions, including input data normalization, CNN layer selection, pixel balancing in input image labels, and fine-tuning the model’s training options. As a result, the goal of this paper was to design an FCN-based segmentation model for the LV from short-axis MR images, which includes the following contributions:

1. Compare various optimization algorithms and select the most reliable one to train the proposed model;
2. Class weighting method to avoid high imbalance of pixels between object and background classes in image’s labels;
3. Pixel normalization of labels to allow the model to learn and extract features from input images accurately;
4. Achieve state-of-the-art results for automatic LV segmentation.

The rest of this paper is organized as follows: Section 2 describes the related works, the materials and methods are introduced in Section 3, and the experimental results and discussion are presented in Section 4, followed by a conclusion in Section 5.

## 2. Related Works

In recent years, segmentation and quantification of the LV from cardiac MRI images have received much attention to diagnose cardiovascular disease. Many studies have proposed semi-automatic segmentation methods to delineate the LV borders, such as active contour [17,18], level set [19,20,21], graph cut [22], dynamic programming, and atlas-based models. These traditional segmentation methods necessitate user intervention, which is a time-consuming and tedious task. The difference between semi-automatic and fully automatic segmentation is that the latter is better suited to process large batches of cardiac MRI images.

For segmenting the LV and myocardium from CMR images, CNNs in various orders have been proposed. Dangi et al. [23] created a CNN-based multi-task learning (MTL) model for simultaneous LV segmentation and quantification. They used the U-net architecture [24], separating segmentation and regression at the final upsampling layer. This network is capable of learning feature representation while also improving generalization. Moradi et al. [25] developed a deep-learning-based method called MFP-U-net for LV segmentation from echocardiography images, and they designed a network with a feature pyramid that can detect and recognize the LV in MRI. Wu et al. [26] proposed an automatic segmentation model for the LV from cardiac MRI. They used a CNN model to locate the LV and the U-net model to segment it. Abdeltawab et al. [10] devised a framework that begins with FCN-based localization of the LV and extraction of the heart section’s ROI. The extracted ROIs are then fed into the FCN2 network, which segments the LV cavity and myocardium. Dong et al. [27] proposed a CNN-based model with two parallel subnetworks to detect endocardium and epicardium contours of the LV, incorporating the MTL concept. The FCN [28] is a CNN expansion with different last layers used for different tasks. Traditional CNN methods, for example, use fully connected layers for image classification to predict objects, whereas an FCN applies a deconvolution (transposed) layer instead of a fully connected layer in semantic segmentation. Several FCN-based models have been used to improve LV segmentation performance [29,30,31]. The network proposed by Cui et al. [32] was an attention U-Net model based on an FCN structure for cardiac short-axis MRI segmentation. U-Net [24] has been commonly applied in medical image segmentation, particularly in the segmentation of cardiac images [25,33,34].

Some researchers used a hybrid model that combined deep learning methods with traditional models to achieve an optimal LV segmentation performance from short-axis cardiac MRI images. For example, Ngo et al. [35] used a deep learning model combined with a level set for automatic LV segmentation. Avendi et al. [36] developed a fully automatic segmentation model for the LV using deep learning algorithms and deformable models. Due to the strong correlation between sequential frames during the cardiac cycle, a 3D model with a recurrent neural network (RNN) has been proposed. Long short-term memory (LSTM) is a popular RNN [37] technique for detecting heart motion using spatiotemporal dynamics. Zhang et al. [38] created a multi-level LSTM model for LV segmentation that used low-resolution level features to train one model and high-resolution level features to train another. Additionally, due to the large slice thickness, Baumgartner et al. [39] found that segmentation by 2D CNN performed better than 3D CNN. Furthermore, due to significant morphological differences in LV shape across slices caused by heart movement, RNN models reproduce incorrect features and require high computational costs. Bernard et al. [40] conducted a benchmark study and discovered that FCNs are used in most advanced algorithms for LV segmentation from short-axis MRI images.

In recent years, researchers have been paying more attention to the segmentation of LV boundaries (endo- and epicardium) from short-axis MRI images. Table 1 summarizes the most recent studies in LV segmentation from short-axis MRI using deep learning models. Furthermore, the LV segmentation challenges [40,41,42] and benchmark datasets with ground truth contours are provided. Deep learning methods have lately obtained excellent results in the segmentation of medical images. CNN is one of the most widely used methods in medical image analysis [23,43] among these approaches. Medical images are segmented at the pixel level, as opposed to image-level classification [27]. Traditional CNN methods must be improved in order to achieve robust semantic segmentation. Furthermore, according to recent research, image pixel class imbalance can affect CNN performance during classification and segmentation [44]. Buda et al. [45] provided a thorough analysis of the CNN class imbalance problem. Data-level methods and classifier methods are two types of solutions to this problem. Oversampling [46] and data augmentation [47] are data-level methods that work with training datasets, whereas classifier-level methods such as cost-sensitive learning [48], hard mining [49], and loss function work with model training options.

Pixel imbalance between the target class and the background class has a significant effect on segmentation performance, which requires an effective solution. Hence, various methods have been proposed to deal with this issue; for example, the focal Tversky loss function (FTL) was introduced by Cui et al. [32], Dong et al. [27] applied cross-entropy loss function instead of the dice loss function, and Wang et al. [15] used dynamic pixel-wise (PW) weighting. In addition, the authors normalized the pixel intensity of the input images to improve the learning ability of the models. Cui et al. [32] used mean–variance normalization (MVN) to normalize the pixel intensity on an input image by subtracting the difference from its average value and dividing by its standard deviation, and Wang et al. [15] used min-max normalization. Based on the above literature, in this study we created a 2D FCN technique with fewer parameters for accurately segmenting the LV and myocardium from short-axis MRI images. After using appropriate normalization and conversion techniques, the input images were used to extract pixels. The 2D PNG images have some advantages compared with NIfTI images, such as flexible image visualization, augmentation (rotation, cropping, and rescaling), and efficient exclusion of unwanted images.

## 3. Materials and Methods

### 3.1. Task Description

**Figure 2:**
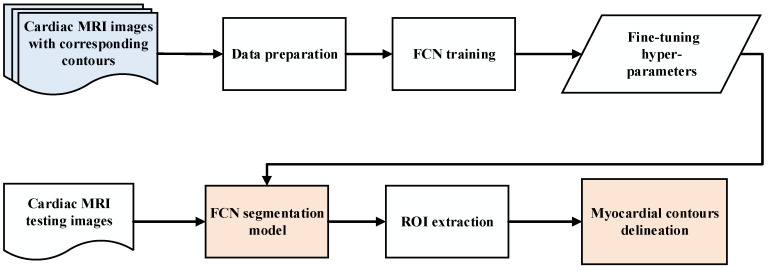
Diagram of the proposed model.

The procedures of the proposed system for LV segmentation are shown in Figure 2. The steps of the system are as follows: (i) preparation of the MRI images, including resizing and pixel normalization; (ii) training the FCN model with a comparison between three optimization algorithms, namely stochastic gradient descent (SGD), adaptive moment estimation (Adam), and root mean square propagation (RMSProp); and (iii) testing the trained model for extraction of ROI features and segmentation to delineate the LV contours.

### 3.2. Data Description

**Figure 3:**
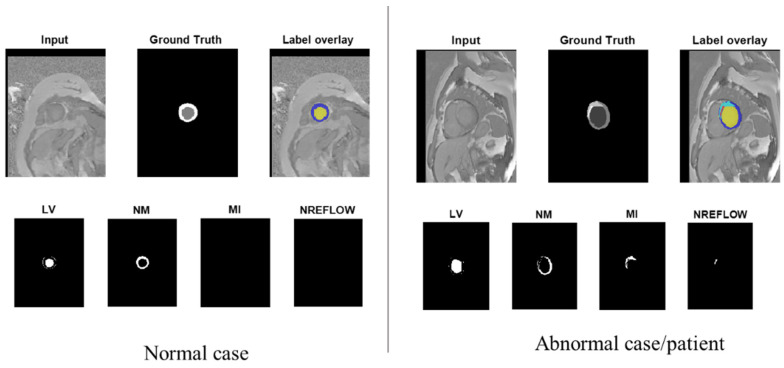
MR images’ visualization from EMIDEC dataset.

The dataset for this study was acquired at the University Hospital of Dijon in France and was provided from the automatic evaluation of myocardial infarction from delayed-enhancement cardiac MRI (EMIDEC) [42] during the MICCAI conference 2020. This dataset contains sequences of short-axis MRI images with ground truth for 150 patients (100 for training and 50 for testing). Each case has a text file with clinical information, a neuroimaging informatics technology initiative (NIfTI) file with the short-axis images of the LV, and an NIfTI file with the labeled masks. The masks consist of four different pixels for each area, which are 0, 1, 2, 3, and 4, representing the background, LV cavity, normal myocardium (NM), myocardial infarction (MI), and no-reflow (NREFLOW), respectively, as shown in Figure 3. The dataset can be downloaded from the website (http://emidec.com/ (accessed on 1 December 2021)). 

### 3.3. Data Preparation

Medical images are commonly stored in NIfTI or DICOM format after being acquired from medical imaging modalities. Although these formats have high precision for images, they provide volumetric (voxels, height, and depth) data with unequal depth in various slices/series. Furthermore, image preprocessing steps such as augmentation and excluding unwanted images from apical slices are quite tedious with volumetric data (3D). Thus, in this study, the NIfTI data were converted to 2D images (PNG) as inputs to train the proposed 2D FCN. 

#### 3.3.1. Data Conversion and Normalization 

Data were converted from NIfTI to PNG images with extensive consideration of the pixels representation. An open-source toolkit named XMedCon was used for medical image conversion [54]. This platform is a graphical user interface (GUI) that gives immediate visual control on selected options with various features, including simple image processing, volume manipulation, pixel values support, and supporting image formats for all medical modalities. The principle of this toolkit is to preserve data and assure that the default output represents the pixel data as retrieved from the original study. This initial step has achieved a more desirable performance for adequate pixel representation than direct conversion by programming code in MATLAB or Python.

**Figure 4:**
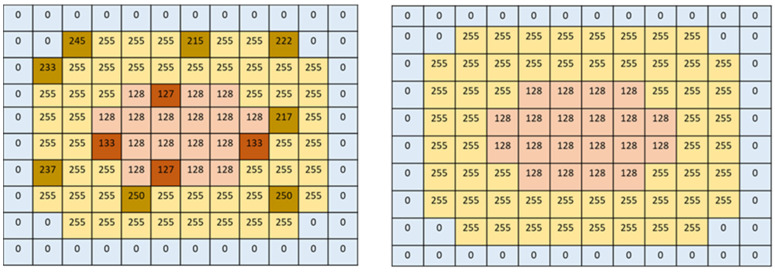
Pixels of image labeled by usual conversion (**left**) and conversion by XMedCon (**right**).

The pixels for the LV and myocardium after image conversion and pixel normalization are depicted in Figure 4, showing the robustness of the conversion step in the right image, whereas the left image represents image pixels using the usual conversion method. The size of all input images was 256 × 192 pixels, with normalized pixel intensities from [1, 2] to [128, 255] for the LV and myocardium, respectively. The pixel normalization ($N_{P}$) was applied using the following equation:

(1) $$N_{P}=\;255\;\times \;mat2gray\left(N_{ori}\right)$$

where $N_{ori}$ represents the pixels matrix of the original image.

#### 3.3.2. Balancing of Class Weight Pixels

Most pixels in the ground truth (labels) are for background, leading to class imbalance. During the learning process, network biases to learning the dominant class (background) result in weak segmentation performance. The balancing of pixels for three classes, including background (BG), LV, and myocardium (Myo), is presented. The class weighting method was used to compute class weights, namely inverse frequency weighting, where the weights of classes are the inverse of the class frequencies.

### 3.4. Network Architecture

**Figure 5:**
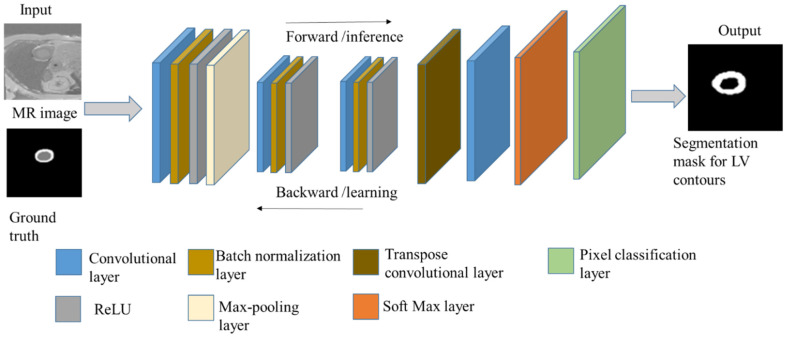
Architecture layers of the proposed FCN.

**Figure 6:**
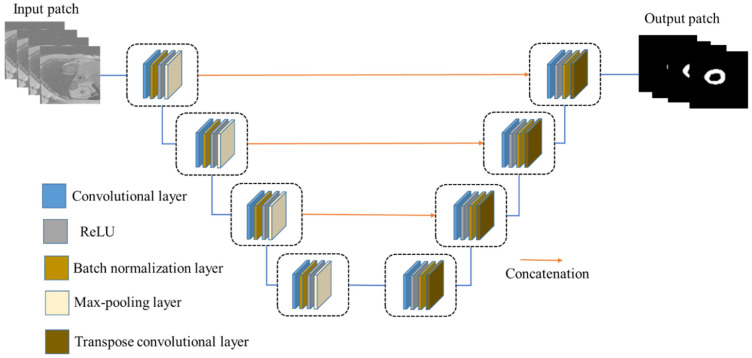
Schematic architecture of the U-Net model.

The first step in creating a new FCN is to define and select suitable layers. Figure 5 depicts the proposed FCN architecture with input and output images. This network takes the principle of U-Net architecture, which has encoder (contraction/downsampling) and decoder (expansion/upsampling) paths, as shown in Figure 6. The contraction path extracts local features and restores feature maps in the expansion path of the network. The network is designed to train a few samples. The layers of the downsampling path are 3 × 3 convolutions, batch normalization, and a rectified linear unit (ReLU) as an activation function with padding to keep the output size of the convolution layer the same as its input. Then, there is downsampling followed by max-pooling operation with a size of 2 × 2 and stride of 2 to reduce the input size. The 4 × 4 transpose convolution (deconvolution) and convolution layers are applied in the upsampling path followed by a pixel classification layer with a softmax layer to predict the output image. The cross-entropy term is used as a loss function in this network.

After defining the layers of the FCN model, the next step is setting up the training options for the network by specifying some parameters such as the solver, the maximum number of epochs, and the learning rate. Solvers such as SGD, Adam, and RMSProp update the network parameters using a subset of data at each step called a mini-batch to minimize the loss function. The parameter updating is named an iteration while the epoch passes through the entire data during network training. The learning rate is a crucial parameter for network training that can shorten the training time and minimize the loss in training progress.

During training, the network performs a forward pass, where each layer takes the output from the previous layer as the input and then outputs the results to the next layer, and a backward pass, where each layer takes the derivative of the loss concerning the layer’s outputs and computes it to the inputs to propagate the results. At the end of the forward pass, the network output layer calculates the loss *L* between the target *T* and the prediction *Y*. Table 2 illustrates the layer types of the FCN model with their kernel sizes and learnable parameters, such as bias and weights. The advantage of the proposed FCN is that its training time is faster and it requires less memory space than U-Net, which requires much time for training and consists of many parameters that need a high computational cost.

In this work, the proposed network was trained with three different optimization algorithms to select one after comparing their performances and efficiency. Moreover, the hyper-parameters of the training options, such as epochs, mini-batch size, and learning rate, were fine-tuned through sequent experiments to select desirable parameters for the proposed network. Based on the experiments, the optimal mini-batch sizes for normalized images were 4 and 8 due to the data size and to lessen the memory space. Thus, the selection of mini-batch size was decided, which evaluated the gradient of the loss function and updated the weights significantly, resulting in a considerable performance of the network. The performance evaluation of this work was determined using metrics such as sensitivity, specificity, negative predictive value (NPV) and positive predictive value (PPV), Jaccard index, and dice score coefficient (DSC).

## 4. Results and Discussion

The proposed method was implemented using MATLAB software (version r2020b) with an Intel (R) i7-3770 central processing unit (CPU), 20 GB DDR3 random access memory (RAM), and Nvidia GeForce GTX 1050 Ti. The initial experiments were conducted to select the hyper-parameters used for the training of the proposed network. After that, the network was trained to segment the LV from MRI images based on the proposed conversion method for the images.

### 4.1. Hyper-Parameters Selection

**Figure 7:**
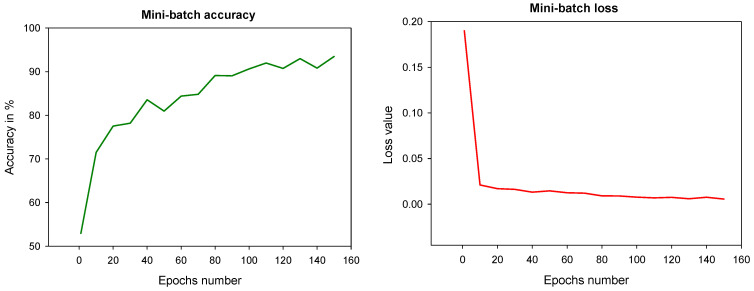
The mini-batch accuracy and mini-batch loss when training the proposed network.

These experiments aimed to select hyper-parameters for the network training, including optimization algorithms (SGDM, Adam, and RMSProp), learning rate, epochs, and mini-batch size. The algorithms’ performance was compared using learning rates of 0.01 and 0.001 over 30, 50, 100, and 150 epochs at mini-batch sizes of 4 and 8. As shown in Table 3 and Table 4, the Adam algorithm achieved the highest performance using a learning rate of 0.001, 150 epochs, and a mini-batch size of 4. Based on the hyper-parameters selection, the proposed FCN was trained and gained a mini-batch accuracy of 91.18% and mini-batch loss of 0.005, as illustrated in Figure 7. Hence, it is evident that the network’s segmentation performance can be improved by using fewer mini-batches and increasing the number of epochs.

### 4.2. Network Performance

**Figure 8:**
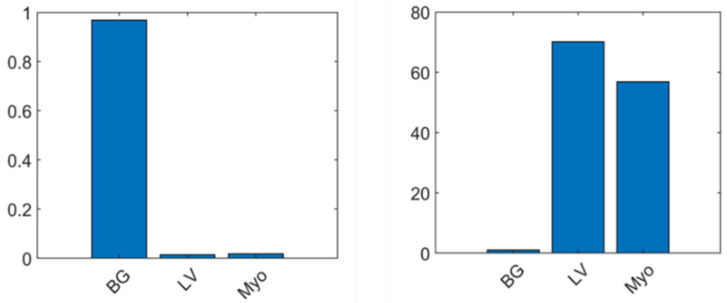
Pixels of classes before balancing (**left**) and after balancing (**right**).

**Figure 9:**
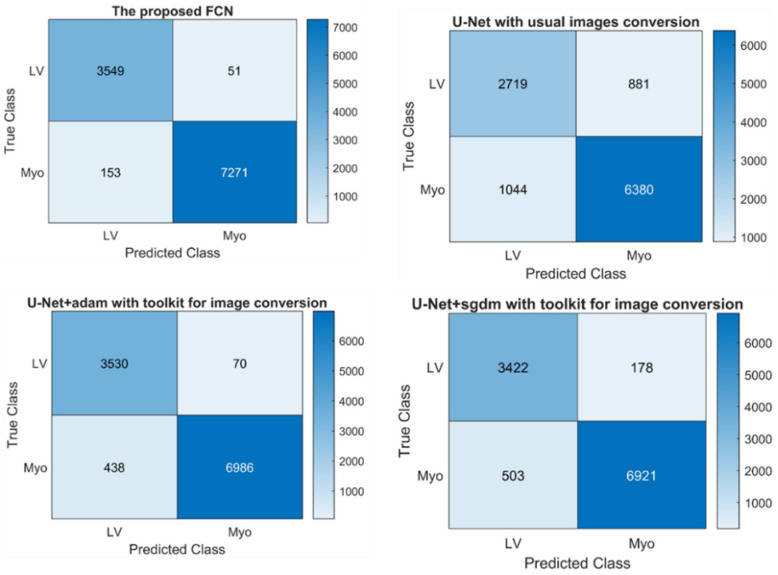
Confusion matrices of the trained models.

The image conversion using the XMedCon toolkit and pixel normalization with pixels weight balancing resulted in an improved performance. The comparison of evaluation metrics between the FCN model and U-Net models based on the proposed image conversion and normalization is shown in Table 5. The proposed FCN model outperformed U-Net models in terms of Jaccard index, sensitivity, NPV, and dice similarity with scores of 0.87, 0.98, 0.99, and 0.93, respectively. The U-Net model with the Sgdm solver performed well only in specificity and PPV, obtaining 0.98 for both. The performance of U-Net without data conversion by the XMedCon toolkit was the worst among the four models, with less efficiency in minimizing loss function based on data features. Furthermore, as depicted in Table 6, the proposed model outperformed the other trained models in terms of global and mean accuracies, mean intersection over union (IoU), weighted IoU, and mean boundary F1 (BF) score, with values of 0.95, 0.96, 0.90, 0.91, and 0.89, respectively. The results shown in Table 5 and Table 6 prove that models using images converted by the XMedCon toolkit as input perform well. Although, the exact features representation of pixels after conversion, an imbalance of target and background pixel classes was found. Thus, pixel weight balancing of the background, LV, and myocardium (Myo) classes was applied and achieved high balancing of the pixels as shown in Figure 8. The confusion matrices used to refine the trained and proposed models are depicted in Figure 9 with prediction of classes’ pixels of the LV and myocardium.

**Figure 10:**
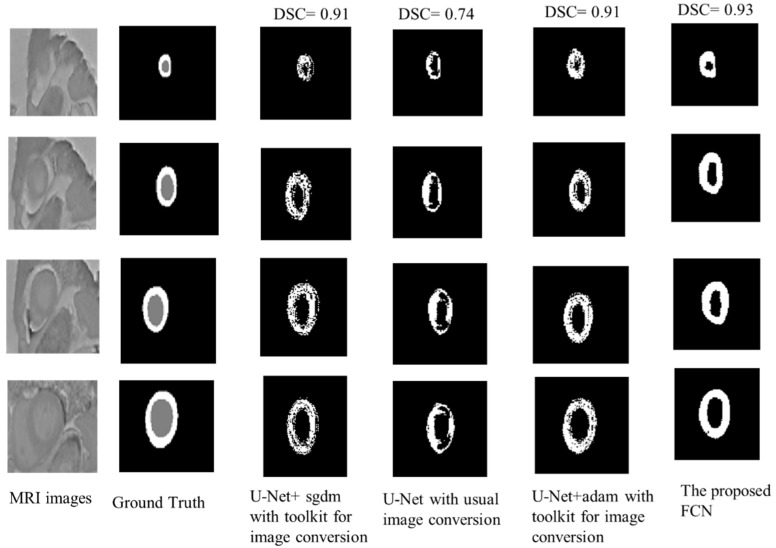
Segmentation results from comparison between U-Net models under various conditions and the proposed FCN.

Figure 10 shows a visual comparison of the output for LV labels using the selected model (with LR = 0.001, epoch = 150) and other models with the referenced ground truths. The proposed model’s segmentation results are very close to the expert-provided LV boundaries. Furthermore, the figure compares the segmentation results obtained from the proposed model with pre-trained U-Net models under various conditions. The proposed FCN model outperformed other methods for delineating LV contours.

### 4.3. Comparison with Recent Methods

The quantitative comparison of the LV segmentation results between the proposed model and other advanced methods is depicted in Table 7. These methods include the attention U-Net architecture [32], convolutional neural network regression (CNR) method [50], FCN method [51], multi-scale FCN DenseNet [8], and a dynamic pixel-wise weighting-based FCN [15]. The detailed datasets and data preparation steps for these models are presented under the related work sections in Table 1. It can be observed that the proposed method achieved a robust performance compared to other published methods. For most evaluation metrics, including the Jaccard index, sensitivity, PPV, NPV, and DSC, the proposed FCN model outperformed other methods, except for the specificity of the method proposed by Wang et al. [15]. It is important to know that Wang’s method involves a dynamic pixel-wise weighting technique to adjust the pixel’s weight according to the upper layer’s segmentation accuracy and forces the pixel classifier to consider the misclassified ones. The network’s performance was based on specific data of a hundred images that underwent normalization and manipulation to be suited for the trained network. The network was tested for ten normal subjects on the same training dataset and had the most significant advantage of being trained using a small normalized dataset.

To the best of our knowledge, LV segmentation is essential to evaluate cardiac function by measuring parameters such as LV volume, LV mass, and ejection fraction. The results show that the performance of the proposed method to delineate the LV contours is very close to the ground truth provided by clinical experts. Thus, on high-contrast images, the proposed network obtains intelligible results allowing doctors to detect cardiac diseases such as myocardial infarction precisely based on automatic LV segmentation. 

### 4.4. Limitation of the Study

The size of the endocardial and epicardial regions from LV segmentation in apical slices was not always accurate compared with basal and middle slices. The main limitation in this study is the number of datasets that need to be enlarged and appropriately configured to train FCN models. In addition, the setting up of significant parameters for network training requires more training data with augmentation.

## 5. Conclusions

In this paper, an FCN was proposed for LV segmentation from short-axis MRI. The selection of training hyper-parameters, such as optimization algorithm, epoch’s number, learning rate, and mini-batch size, was based on multiple experiments training various model structures. The input images used for the model were initially converted using a toolkit that keeps the feature representation of pixels the same as the original data. Data normalization in this study performed well and allowed the network to learn feature extraction accurately. In addition, pixel weighting was introduced to avoid an imbalance in target class and background class pixels. Overall, the proposed network has achieved a robust performance in terms of Jaccard index, dice metric, sensitivity, specificity, PPV, and NPV, which is a significant step towards reducing manual segmentation by clinical experts during the diagnosis of cardiac diseases.

## Figures and Tables

**Table 1:** Current studies in LV segmentation from cardiac MRI using deep learning algorithms.

Author/Year	Dataset	Subjects No.	Data Preparation	Deep Learning Model
Cui et al. [32]/2021	LVSC	200	- Cropping using multi-scale methods - Pixel normalization	Attention U-net architecture
Tan et al. [50]/2017	LVSC	200	- Resampling pixels using linear interpolation - Cropping - Pixel normalization - Augmentation (during training)	CNR
Tran et al. [51]/2016	SCD, LVSC and RVSC	45, 200 and 48	- Cropping using multi-resolution approach - Augmentation	FCN
Khend et al. [8]/2019	ACDC-2017, LVSC and Kaggle	150, 200 and 500	- Cropping - Augmentation	FCN DenseNet
Wang et al. [15]/2020	CAP	450	- Cropping - Augmentation - Pixel normalization	FCN
Wu et al. [26]/2020	SCD	45	- Image filtering - Cropping/downsampling	CNN + U-Net
Wu et al. [52]/2021	SCD	45	- Augmentation	GAN
Dong et al. [27]/2020	MICCAI 2019	56	- Pixel normalization	Parallel CNNs
Du et al. [53]/2019	2900 collected images	156	- Cropping - Normalization	Multi-task CNR + RNN
Abdeltawab et al. [10]/2020	ACDC-2017	150	- Cropping	Two FCNs

**Table 2:** Analyzing layers of the proposed FCN.

Layer Number	Layer Type	Kernel Size	Learnable
1	Image input	256 × 192 × 1	-
2	Convolution	256 × 192 × 16	Weights 3 × 3 × 1 × 16 Bias 1 × 1 × 16
3	Batch normalization	256 × 192 × 16	Offset 1 × 1 × 16 Scale 1 × 1 × 16
4	ReLU	256 × 192 × 16	-
5	Max pooling	128 × 96 × 16	-
6	Convolution	128 × 96 × 32	Weights 3 × 3 × 16 × 32 Bias 1 × 1 × 32
7	Batch normalization	128 × 96 × 32	Offset 1 × 1 × 32 Scale 1 × 1 × 32
8	ReLU	128 × 96 × 32	-
9	Convolution	128 × 96 × 64	Weights 3 × 3 × 32 × 64 Bias 1 × 1 × 64
10	Batch normalization	128 × 96 × 64	Offset 1 × 1 × 64 Scale 1 × 1 × 64
11	ReLU	128 × 96 × 64	-
12	Transpose convolutional layer	128 × 96 × 16	Weights 4 × 4 × 16 × 64 Bias 1 × 1 × 16
13	Convolution	256 × 192 × 2	Offset 3 × 3 × 16 × 2 Scale 1 × 1 × 2
14	Softmax	256 × 192 × 2	-
15	Pixel classification layer	-	-

**Table 3:** Optimization algorithms’ performance in the trained network at mini-batch size 4.

Learning Rate	Epochs	SGDM %	ADAM %	RMSProp %
0.01	30	74.67	60.18	50.25
	50	76.41	54.39	54.85
	100	82.45	51.34	65.11
	150	76.81	60.69	47.22
0.001	30	70.68	78.91	81.76
	50	74.18	81.27	83.82
	100	77.27	87.14	85.65
	150	76.92	91.18	89.20

**Table 4:** Optimization algorithms’ performance in the trained network at mini-batch size 8.

Learning Rate	Epochs	SGDM %	ADAM %	RMSProp %
0.001	30	60.63	79.85	78.00
	50	69.00	81.17	81.69
	100	70.65	87.60	81.96
	150	60.37	90.42	87.44

**Table 5:** Comparison of evaluation metrics between trained models and the proposed FCN model (√ represents conversion by XMedCon and × represents conversion by coding).

Model	Conversion by XMedCon	Jaccard Index	Sensitivity	Specificity	PPV	NPV	DSC
U-Net + sgdm	√	0.84	0.86	0.98	0.98	0.89	0.91
U-Net	×	0.60	0.78	0.85	0.72	0.89	0.74
U-Net + adam	√	0.84	0.93	0.91	0.89	0.94	0.91
The proposed FCN	√	0.87	0.98	0.94	0.89	0.99	0.93

**Table 6:** Performance results of the trained models (√ represents conversion by XMedCon and × represents conversion by coding).

Model	Conversion by XMedCon	Global Accuracy	Mean Accuracy	Mean IoU	Weighted IoU	Mean BF-Score
U-Net + adam	√	0.93	0.92	0.86	0.86	0.89
U-Net	×	0.83	0.82	0.69	0.71	0.67
U-Net + sgdm	√	0.92	0.92	0.85	0.85	0.85
Our FCN	√	0.95	0.96	0.90	0.91	0.89

**Table 7:** Performance comparison between the proposed model and other state-of-the-art models in automatic LV segmentation.

Method	Jaccard Index	Sensitivity	Specificity	PPV	NPV	Dice
Cui et al. [32]	0.75	0.87	0.92	0.87	0.93	-
Tan et al. [50]	0.77	0.88	0.95	0.86	0.96	-
Tran et al. [51]	0.74	0.83	0.96	0.86	0.95	-
Khend et al. [8]	0.74	0.84	0.96	0.87	0.95	0.84
Wang et al. [15]	0.70	0.90	0.99	0.77	0.99	0.80
The proposed FCN	0.87	0.98	0.94	0.89	0.99	0.93

## Data Availability

The data presented in this study are available on request from the corresponding author.
